# Corrugator Supercilii Muscle and Its Relationship with Neurovascular Structures of the Frontal Region: A Cadaveric Study

**DOI:** 10.1007/s00266-026-05933-w

**Published:** 2026-05-26

**Authors:** Nilay Yildiz, Gkionoul Nteli Chatzioglou, Osman Coşkun, Ayşin Kale, Özcan Gayretli

**Affiliations:** 1 Department of Anatomy, Faculty of Medicine, Istanbul University, Istanbul, Türkiye; 2 Department of Anatomy, Faculty of Medicine, Alanya Alaaddin Keykubat University, Antalya, Türkiye; 3 Department of Anatomy, Faculty of Medicine, Istanbul Health and Technology University, İstanbul, Türkiye

**Keywords:** Corrugator supercilii muscle, Supratrochlear nerve, Supraorbital nerve, Migraine surgery, Botulinum toxin

## Abstract

**Background:**

The corrugator supercilii muscle (CSM) is a critical anatomical target for botulinum toxin injections and surgical interventions in migraine treatment and facial rejuvenation. However, complications such as sensory loss or vascular injury may arise due to its proximity to neurovascular structures. This study aims to delineate the precise anatomy of the CSM and its relationship with the supratrochlear (STN) and supraorbital nerves (SON) to enhance procedural safety.

**Methods:**

A cadaveric dissection was performed on 44 hemifaces of 22 formalin-fixed cadavers (11 male and 11 female). The CSM and adjacent neurovascular structures were dissected, photographed, and measured using ImageJ software. Morphometric parameters included distances related to the CSM, as well as branching patterns of the STN and SON were evaluated.

**Results:**

The CSM length was significantly longer in females (30.53 ± 2.60 mm; 28.93 ± 2.15 mm, *p* = 0.032). The STN exhibited complex branching (> 3 branches) in 48% of cases, with 13.6% piercing the CSM anteriorly. The SON medial branch pierced the CSM’s lateral segments (46.5% 3rd segment; 53.5% 2nd segment). The supratrochlear artery crossed the CSM 14.84 ± 2.05 mm (females) and 15.43 ± 2.04 mm (males) from the midline, while the supraorbital artery lay 25.38 ± 3.76 mm (females) and 24.49 ± 4.55 mm (males) lateral to the midline.

**Conclusions:**

The CSM’s intimate relationship with the STN, SON, and associated vessels underscores the risk of iatrogenic injury during forehead procedures. Anatomical variations in nerve branching and muscle morphology highlight the need for individualized approaches to botulinum toxin injection and surgical resection. These findings may reduce complications and improve outcomes in migraine surgery and aesthetic interventions.

**No Level Assigned:**

This journal requires that authors assign a level of evidence to each submission to which Evidence-Based Medicine rankings are applicable. This excludes Review Articles, Book Reviews, and manuscripts that concern Basic Science, Animal Studies, Cadaver Studies, and Experimental Studies. For a full description of these Evidence-Based Medicine ratings, please refer to the Table of Contents or the online Instructions to Authors www.springer.com/00266.

## Introduction

The muscles in the frontal region, eyebrows, and around the eyes have an important role in communication by creating emotions and facial expressions. The position of the eyebrows is a tool to express a person's mood [1]. The facial region has been an important intervention site for dermocosmetic applications from the past to the present. Wrinkles formed between and above the eyebrows, mainly due to the activation of the corrugator supercilii muscle, procerus muscle and depressor supercilii muscle in this region, increasing patient applications due to aesthetic concerns. The procedures of this region are performed by contracting the corrugator supercilii muscle and injecting botulinum toxin from reference points determined according to this muscle [2].

Many migraine and primary headache types have been associated with compression or entrapment of peripheral nerves at trigger points [3]. Sensory innervation areas of the supraorbital and supratrochlear nerves, located in the frontal region, are among these trigger points [4]. It has been shown that there is a significant decrease in the frequency and severity of migraine headache attacks by injection of botulinum toxin into the muscles in the trigger areas [5–7]. The anatomical shape of the points through which neurovascular structures pass is effective in the etiology of nerve compression. Information about the compression of the supraorbital nerve by fascial bands in the supraorbital notch or while passing through the supraorbital foramen is available in the literature [4, 8]. There are also studies showing that hypertrophy of the corrugator supercilii muscle is involved in the etiology [9]. Based on the effectiveness of botulinum toxin application, it was thought that a successful treatment model would be created when the muscle responsible for the trigger point was surgically removed. This idea formed the basis of migraine surgery, which is becoming increasingly common nowadays [3, 5]. It has been reported that in cases such as rejuvenation operations or migraine surgery in which the corrugator supercilii muscle is surgically removed partially or completely, sensory loss in the forehead area can be expected due to the close relationship of the supratrochlear and supraorbital nerves with the muscle [10].

Considering the frequency and necessity of such clinical applications, the aim of our study is to elucidate the neuromuscular structure and neighborhood of the region in question and to report the sensory innervation and vascularization properties of the corrugator supercilii muscle.

## Materials and Methods

The study included 44 sides of 22 formalin-fixed cadavers (11 females and 11 males) aged between 18 and 88, in the Anatomy Department of Istanbul Faculty of Medicine. Ethics committee approval was received from the Istanbul Faculty of Medicine Clinical Research Ethics Committee dated 22.07.2024 and numbered 2736236, and the study process was carried out in accordance with the 1964 World Medical Association Helsinki Declaration.

Cadavers with preserved frontal and orbital region integrity were included in the study.

Exclusion criteria were as follows:

- Cases with previous surgery,
- Cases with trauma or a mass
- Cases with orbital integrity impaired were excluded from the study.

After the dissection procedures of the cadavers were carried out, they were photographed at every stage using a millimetric ruler, and these photographs were transferred to digital media. While several studies have shown that mobile device cameras can be safely used for data transfer when supported by appropriate applications [11], in our study, a conventional paper metric was preferred for photographing in accordance with cost considerations, physical feasibility, and data security, as the data were transferred to a digital medium for processing.

Measurements were made with the obtained images using the ImageJ 1.54g Java 13.0.6 (National Institutes of Health, USA) program. Measurement results were recorded using the Microsoft Excel program.

### Statistical Analysis with Jamovi Computer Software

In our study, descriptive statistics were used to demonstrate the general distribution of morphometric measurements, and all statistical analyses were conducted using the Jamovi software (The jamovi project (2024). (Version 2.6) (Computer Software). Retrieved from https://www.jamovi.org).

A significance level of *p* < 0.05 was accepted for all analyses. To examine relationships between parameters, correlation analysis was performed, assessing the strength and direction of linear relationships between two morphometric variables using Pearson’s correlation coefficient (r). To determine whether there was a statistically significant difference between the right and left side measurements of the same cadavers, a Paired Samples *t* test was applied. When significant differences (*p* < 0.05) were found, effect size was evaluated using Cohen’s d, where values above 0.8 indicated a large effect, values between 0.5 and 0.8 a moderate effect, and values below 0.2 a very small effect. Additionally, an Independent Samples *t* test was used to compare male and female groups, and significant differences were again assessed using Cohen’s d to determine the magnitude of the effect.

A significance level of *p* < 0.05 was accepted for all analyses. The use of parametric tests (Paired Samples and Independent Samples *t* tests) was justified based on the properties of morphometric data, which typically follow a normal distribution, and the central limit theorem, which supports their use with sample sizes exceeding 30 observations. Explicit normality testing (e.g., Shapiro–Wilk) was not performed as the primary focus was on mean comparisons for which t tests are robust, especially with our sample characteristics.

### Dissection Protocol

**Fig. 1:**
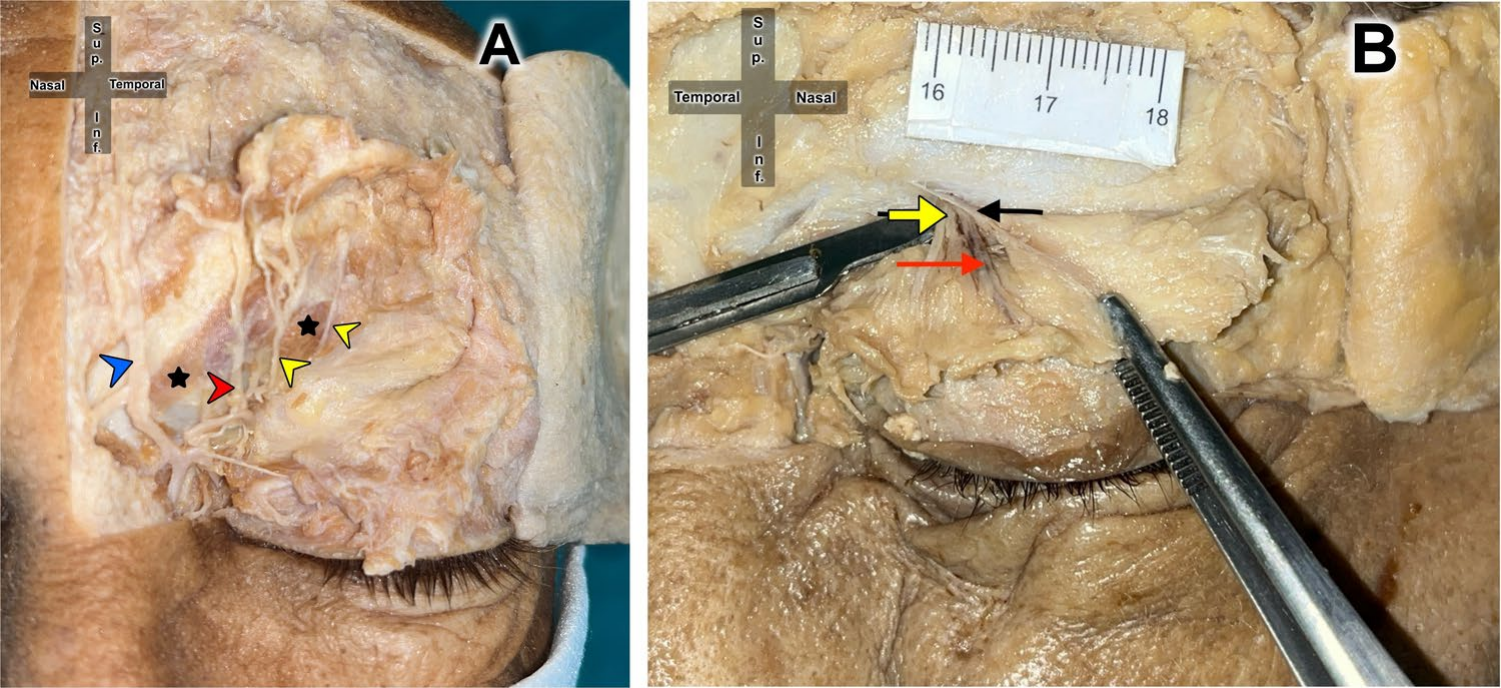
Demonstration of the corrugator supercilii muscle with superficial and deep neurovascular structures. The superficial neurovascular structures around the muscle (**A**) (black stars: corrugator supercilii muscle, blue arrowhead: supratrochlear vein, red arrowhead: supratrochlear artery, yellow arrow heads: branches of the supratrochlear nerve); the deep neurovascular structures around the muscle (**B**) (red arrow: supraorbital artery, black and yellow arrows: medial and lateral branches of the supraorbital nerve)

A vertical midline incision was made, starting from the nasion and continuing to the frontal eminence level. The incision line continued from the nasion laterally along the superior palpebra. Then, skin and subcutaneous dissections were performed. By making fine dissections of the frontalis muscle and orbicularis oculi muscle, the corrugator supercilii muscle, and the neurovascular structures on its surface were reached (Fig. 1A). When these steps were completed, the corrugator supercilii muscle was deviated anteriorly and reached the medial and lateral branches of the supraorbital nerve and supraorbital artery (Fig. 1B).

### The Morphometric Parameters Related to the Corrugator Supercilii Muscle and Its Superficial Neurovascular Structures

During the determination of morphometric parameters, important reference points of CSM (the most medial, lateral, and uppermost points) and superficial landmarks were taken into consideration. Midsagittal and horizontal lines were determined by considering the medial palpebral commissures of the right and left sides as superficial landmarks. Accordingly, for the midsagittal line, a vertical line passing through the midpoint between the right and left medial palpebral commissures was determined. For the horizontal line, a transverse line passing through the right and left medial palpebral commissures was marked.

(P1) STN-MLt: Transverse distance between the exit point of STN from the orbital cavity and the midsagittal line

(P2) STA-MLt: Transverse distance between the point where the STA crosses the lower edge of the CSM and the midsagittal line

(P3) CSMm-MLt: Transverse distance between the most medial point of the CSM and the midsagittal line

(P4) CSMm-HLv: Vertical distance between the most medial point of the CSM and the horizontal line

(P5) CSMl- MLt: Transverse distance between the most lateral point of the CSM and the midsagittal line

(P6) CSMl-HLv: Vertical distance between the most lateral point of the CSM and the horizontal line

(P7) CSMs-MLt: Transverse distance between the uppermost point of the CSM and the midsagittal line

(P8) CSMs-HLv: Vertical distance between the uppermost point of the CSM and the horizontal line

(P9) LCSM: Length of the corrugator supercilii muscle

### The Morphometric Parameters Related to the Corrugator Supercilii Muscle and Its Deep Neurovascular Structures

(P10) SONm-MLt: Transverse distance between the point where the medial branch of the SON pierces the CSM and the midsagittal line

(P11) SONl-MLt: Transverse distance between the point where the lateral branch of the SON passes behind the CSM and the midsagittal line

(P12) SOA-MLt: Transverse distance between the point where the SOA passes behind the CSM and the midsagittal line

### Morphological Evaluation of the Corrugator Supercilii Muscle and Its Neurovascular Structures

The supratrochlear nerve was categorized into three different groups based on its branching pattern: (a) single branch, (b) intermediate branching (1–3 branches), and (c) complex branching (> 3 branches). The number of branches of the supratrochlear and supraorbital nerves at the exit of the orbital cavity and their relationship to the CSM muscle were also observed. To determine this, the CSM muscle was first divided into three equal segments perpendicularly based on the direction of the muscle fibers. These segments were designated segments I, II, and III, from medial to lateral to the muscle. Subsequently, the segment (1st, 2nd, and 3rd segments) of the CSM muscle in which the more superficial supratrochlear nerve coursed was observed and recorded. The segment of the supraorbital nerve, which pierces the CSM inferiorly, was recorded at the point where it pierces the CSM.

## Results

**Fig. 2:**
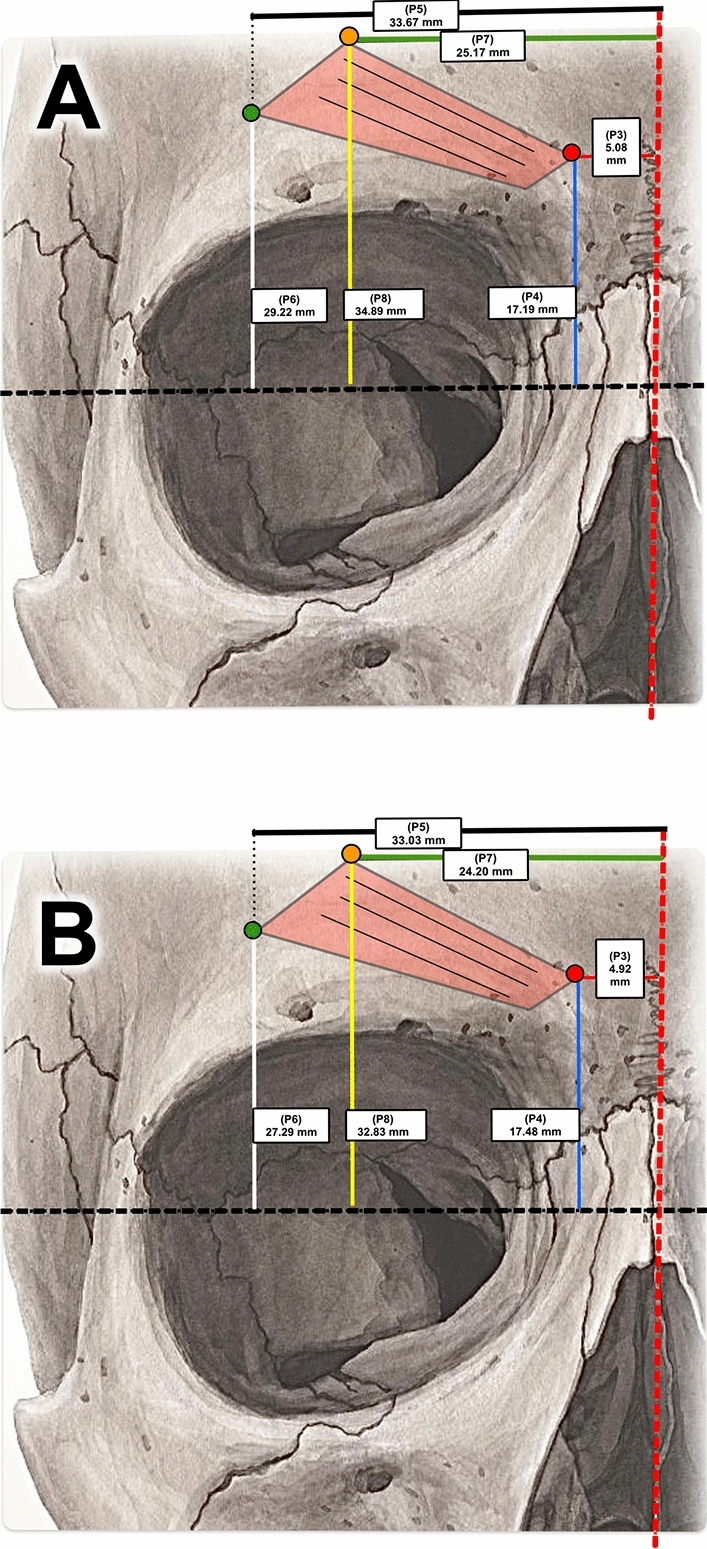
Mean of distances between the corrugator supercilii muscle and the midsagittal/horizontal line in females (**A**) and males (**B**). Vertical line passing through the midpoint between the right and left medial palpebral commissures (dashed red line), transverse line passing through the right and left medial palpebral commissures (dashed black line), the most medial point of the corrugator supercilii muscle (red dot), the most lateral point of corrugator supercilii muscle (green dot), the uppermost point of corrugator supercilii muscle (orange point), P3 parameter (red line), P4 parameter (blue line), P5 parameter (black line), P6 parameter (white line), P7 (green line), P8 (yellow line)

In our study, the transverse distances (P1 and P2) of the supratrochlear artery and nerves between the midsagittal and horizontal lines for the 44 sides examined were found to be greater in males. However, only the mean distance between the supratrochlear nerve and the midsagittal line (P1) was found to be statistically significant in males (*p* < 0.05) (Table 1). The transverse and vertical distances measured from the medial point of the CSM to the midpoint line were similarly found to be greater in males. However, no statistically significant difference was found between genders for parameters P3 and P4 (*p* > 0.05) (Fig. 2). Unlikely, the mean transverse and vertical distances measured from the lateral point of the CSM to the midpoint (P5 and P6) were found to be greater in females (Fig. 2). However, this difference between genders was not found to be statistically significant. Similarly, the mean transverse and vertical distances between the highest point of the CSM and the midpoint were found to be greater in female cadavers. However, this difference was not found to be statistically significant (*p* > 0.05). The mean length of the CSM was 30.53 ± 2.60 mm in women and 28.93 ± 2.15 mm in men. This difference in muscle length between the sexes was found to be statistically significant (*p* = 0.032). Although the distances between the supraorbital nerve and artery and the midpoint (P10, P11, and P12) were higher in women, this difference between the sexes was not statistically significant (*p* > 0.05) (Table 1).

**Table 1:** Results of measured morphometric parameters ​​(dimensions are in mm, SD: standard deviation)

Parameters (mm)			N	Mean	Median	SD	*p*
STN-MLt (P1)	Gender	Female	22	14.47	14.46	1.97	< 0.001*
		Male	22	16.95	16.64	2.17	
	Side	Right	22	15.46	15.20	2.30	0.301
		Left	22	15.96	15.84	2.53	
STA-MLt (P2)	Gender	Female	22	14.84	14.68	2.05	0.343
		Male	22	15.43	15.41	2.04	
	Side	Right	22	14.50	14.41	1.85	0.035*
		Left	22	15.77	16.26	2.07	
CSMm-MLt (P3)	Gender	Female	22	5.08	4.75	2.07	0.778
		Male	22	4.92	4.39	1.63	
	Side	Right	22	4.79	4.94	1.51	0.300
		Left	22	5.21	4.33	2.15	
CSMm-HLv (P4)	Gender	Female	22	17.19	17.02	1.18	
		Male	22	17.48	17.39	1.40	
	Side	Right	22	17.44	17.25	1.23	
		Left	22	17.24	17.20	1.36	
CSMl-MLt (P5)	Gender	Female	22	33.67	33.77	2.58	0.457
		Male	22	33.03	32.72	3.01	
	Side	Right	22	33.00	32.71	3.16	0.360
		Left	22	33.70	33.67	2.37	
CSMl-HLv (P6)	Gender	Female	22	29.22	29.66	2.78	
		Male	22	27.29	27.43	1.66	
	Side	Right	22	28.13	28.17	2.65	
		Left	22	28.39	27.91	2.33	
CSMs-MLt (P7)	Gender	Female	22	25.17	25.58	4.26	0.398
		Male	22	24.20	24.80	3.18	
	Side	Right	22	23.80	24.16	4.09	0.095
		Left	22	25.56	26.02	3.22	
CSMs-HLv (P8)	Gender	Female	22	34.89	35.27	2.91	
		Male	22	32.83	32.88	1.18	
	Side	Right	22	34.09	33.46	2.79	
		Left	22	33.64	32.92	2.04	
LCSM (P9)	Gender	Female	22	30.53	30.48	2.60	0.032*
		Male	22	28.93	29.09	2.15	
	Side	Right	22	29.98	29.82	2.40	0.422
		Left	22	29.49	29.83	2.62	
SONm-MLt (P10)	Gender	Female	21	23.05	24.06	4.52	0.485
		Male	22	22.13	22.08	4.00	
	Side	Right	21	21.6	21.3	4.48	0.051
		Left	21	23.6	24.4	3.94	
SONl-MLt (P11)	Gender	Female	21	29.22	30.01	3.29	0.056
		Male	21	27.34	27.59	3.51	
	Side	Right	20	27.9	27.2	3.91	0.327
		Left	22	28.6	28.6	3.12	
SOA-MLt (P12)	Gender	Female	21	25.38	25.26	3.76	0.494
		Male	21	24.49	23.89	4.55	
	Side	Right	20	24.39	23.62	4.42	0.342
		Left	22	25.42	25.97	3.91

### Results Related to the Morphological Evaluation of the Corrugator Supercilli Muscle and Neurovascular Structures

**Fig. 3:**
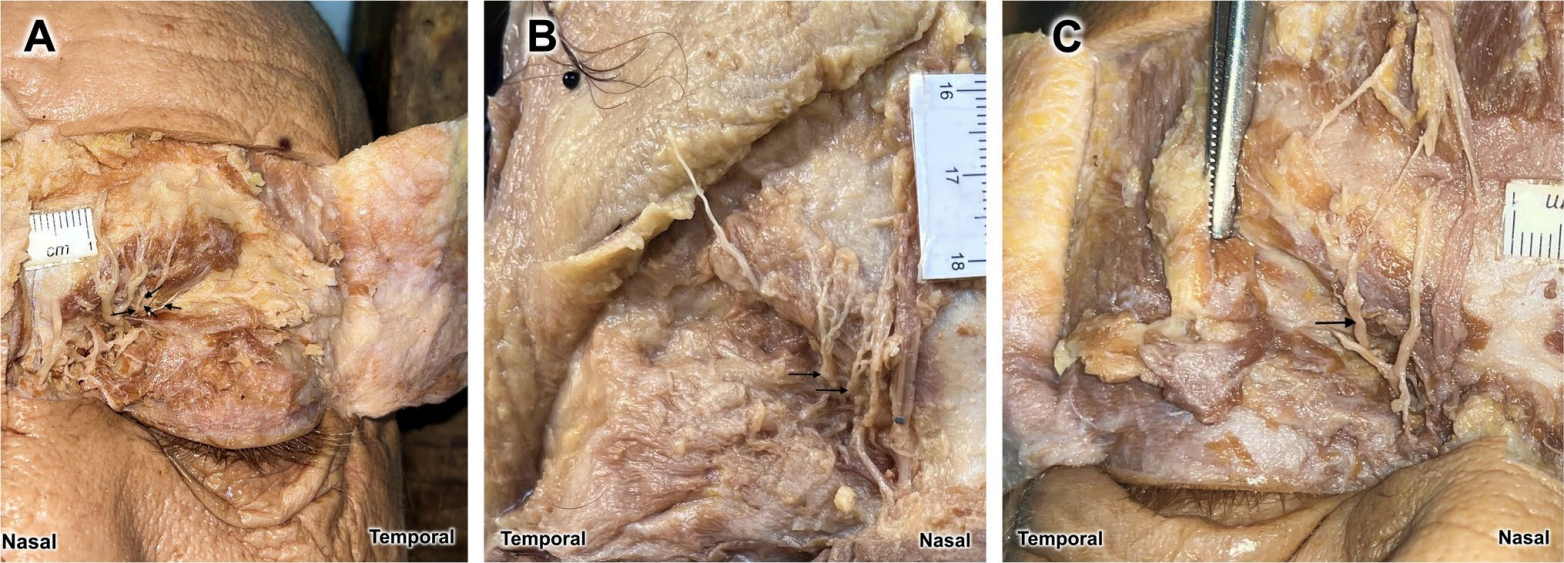
Distribution pattern of the supratrochlear nerve. Complex branching (>3 branches) of supratrochlear nerve (**A**), intermediate branching (1–3 branches) of supratrochlear nerve (**B**), single branch of supratrochlear nerve (**C**). Black arrow(s): supratrochlear nerve(s)

**Fig. 4:**
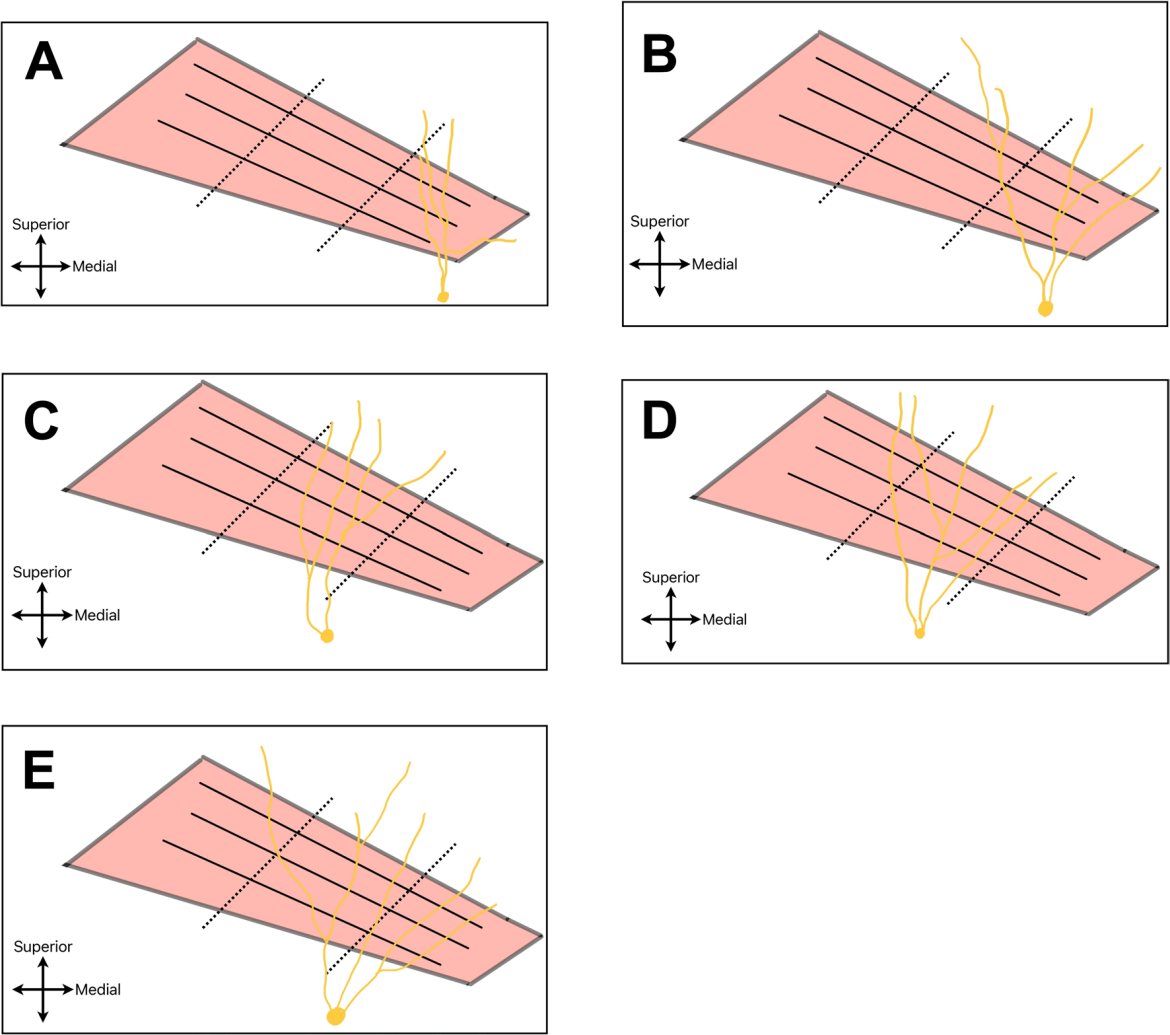
Demonstration of the course of the supratrochlear nerve according to the segments of the corrugator supercilii muscle. The supratrochlear nerve branching anterior to 1st segment of the corrugator supercilii muscle (**A**), the supratrochlear nerve branching anterior to 1st and 2nd segments of the corrugator supercilii muscle (**B**), the supratrochlear nerve branching anterior to 2nd segment of the corrugator supercilii muscle (**C**), the supratrochlear nerve branching anterior to 2nd and 3rd segments of the corrugator supercilii muscle (**D**), the supratrochlear nerve branching anterior to 1st, 2nd, and 3rd segments of the corrugator supercilii muscle (**E**)

The number of branches of the STN as it exited the orbit demonstrated a complex pattern (Fig. 3A) in approximately 48% (21/44) of the sides and an intermediate branching pattern (Fig. 3B) in 32% (14/44). In approximately 20% (9/44) of the cases, it emerged from the orbit as a single branch (Fig. 3C) and then split into branches throughout the frontal region. It was observed that a branch of the STN pierced the CSM from the anterior in a total of 6 sides (13.6%) of 4 cadavers. In addition, the CSM was divided into 3 equal segments according to its length, and the relationship of the STN branches with these segments was evaluated. Accordingly, in 34.3% (15/44) of the cases, the STN traveled only in front of the 1st segment of the muscle (Fig. 4A). In 27.2% (12/44) cases in front of the 1st and 2nd segments of the muscle (Fig. 4B), in 25% (11/44) only in front of the 2nd segment (Fig. 4C), and in front of the 2nd and 3rd segments in 11.2% (5/44) (Fig. 4D). Moreover, it was shown that STN was found irregular in front of the 1st, 2nd, and 3rd segments (Fig. 4E).

The same segmentation process was also applied to determine the point where the SONm pierces the CSM and passes to its anterior surface. It was recorded that SONm pierced the 3rd segment of the muscle in 46.5% (20/43) of the cases, and from the 2nd segment in 53.5% (23/43) of the cases and passed to the anterior surface of the muscle. The SONm could not be shown on one side of one cadaver. It was observed that SONm pierced the muscle from the 2nd segment in 50% and from the 3rd segment in 50% of the male gender. In females, the muscle was pierced from the 3rd segment in 57% of cases and from the 2nd segment in 43% of cases.

## Discussion

### Corrugator Supercilii Muscle

Yang and Kim [12] and Pınar et al. [13] reported data on the oblique and transverse heads of the corrugator supercilii muscle, but Park et al. [14] and Janis et al. [15] stated in their study that no distinction could be made regarding muscle heads. Similarly, in our study, no clear distinction was observed between muscle heads in macrodissections. The literature comparison of our data regarding the most medial, most lateral, and uppermost points of the corrugator supercilii muscle is given in Table 2. Muehlberger et al. [16], in their study examining the corrugator supercilii muscle, reported the length of the muscle to be between 38 and 53 mm. Walden et al. [17] found separate length values for the two heads of the muscle in their study comparing 24 corrugator supercilii muscles in 12 fresh cadavers. Accordingly, the average length of the transverse head of the muscle was reported as 23.38 mm, and the average length of the oblique head was reported as 19.75 mm [17]. In their study on 25 cadavers, Pinar et al. [13] determined the length of the corrugator supercilii muscle as 28.58 ± 7.25 mm on the right side and 29.82 ± 5.74 mm on the left side. In our study, the average length of the corrugator supercilii muscle was found to be 29.98 ± 2.40 mm on the right side and 29.49 ± 2.62 mm on the left side.

**Table 2:** Literature comparison of the distances of the most medial, lateral, and uppermost points of the corrugator supercilii muscle to the midline and horizontal line

Study (year)	N	CSMm–MLt		CSMm–HLv		CSMl–MLt		CSMl–HLv		CSMs–MLt		CSMs–HLv	
		R	L	R	L	R	L	R	L	R	L	R	L
Park et al. [14]	16	6.70	6.70	**–**	**–**	42.70	45.50	**–**	**–**	**–**	**–**	**–**	**–**
Janis et al. [15]	50	2.7 ± 0.9	3.0 ± 1.0	9.8 ± 2.1	9.3 ± 2.0	44 ± 2.6	42.7 ± 3.5	28.9 ± 2.8	26.8 ± 3.5	33. ± 2.8	32.5 ± 2.0	32.8 ± 3.1	31.6 ± 2.5
Yang and Kim [12]	35	4.0 ± 2.2	4.8 ± 1.4	17.5 ± 2.9	17.0 ± 3.7	34.1 ± 3.6	36.7 ± 4.8	27.4 ± 4.8	30.0 ± 4.7	16.5 ± 5.0	16.2 ± 4.7	31.2 ± 3.0	31.2 ± 3.5
Pinar et al. [13]	50	7.43 ± 5.78	7.63 ± 4.08	16.85 ± 2.77	16.29 ± 2.15	37.38 ± 3.02	36.64 ± 5.57	33.11 ± 2.68	33.08 ± 4.19	23.21 ± 4.93	23.09 ± 4.63	32.22 ± 4.72	30.30 ± 7.48
Our Study (2024)	44	4.79 ± 1.51	5.21 ± 2.15	17.44 ± 1.23	17.24 ± 1.36	33 ± 3.16	33.7 ± 2.37	28.13 ± 2.65	28.39 ± 2.33	23.8 ± 4.09	25.56 ± 3.22	34.09 ± 2.79	33.64 ± 2.04

### Supratrochlear and Supraorbital Nerve

When the morphological characteristics of the muscle and the neural structures on its surface are evaluated, it is seen that the data obtained coincide with the study conducted by Isse and Elahi [18] on 10 fresh cadavers. The common findings we have obtained are that the muscle starts from the lateral side of the glabella, with tight attachments to the frontal bone, and ends laterally in the skin by piercing the orbicularis oculi and frontalis muscle, most branches of the supratrochlear nerve run superficial to the muscle, and some branches of the supraorbital nerve pass to the surface by piercing the muscle [18, 19]. Lee et al. [9] reported in their study on 29 cadavers that the average distance between the point where the supratrochlear nerve pierces the CSM and the ML was 16.4 ± 4.0 mm. In our study, the supratrochlear nerve was observed to pierce CSM in only 6 cases. In these cadavers, the transverse distance between the point where the supratrochlear nerve pierces the muscle and the ML was found to be 17.37 ± 4.39 mm.

Shin et al. [20] on 49 sides of 27 fixed cadavers, the average distance between the exit point of the supratrochlear nerve from the orbit and the ML was recorded as 17.1 ± 2.4 mm. In our study, the average transverse distance between the STN-MLt was found to be 15.46 ± 2.30 mm on the right side and 15.96 ± 2.53 mm on the left side.

In recent years, the frequency and scope of plastic surgery interventions have shown a remarkable increase. In particular, the positive and profound impact of reconstructive surgery on human life has become indispensable. Likewise, the significant rise in aesthetic surgical procedures cannot be overlooked. It is evident that in modern surgery, considering the potential risks, complications, postoperative course, and prognosis of both major and minor surgical interventions at all times—and determining the most appropriate approach for patient safety—holds great importance. The advancement of modern technologies has provided substantial support to healthcare professionals in areas such as postoperative wound healing and prognosis. In the literature, there are pilot studies that enable the monitoring of wound measurements and follow-up on a daily, weekly, or monthly basis through mobile phones. These systems assist in predicting prognosis by scoring and analyzing the collected data [11, 21]. In both migraine surgery and facial rejuvenation procedures, complications such as bleeding, deformity, and wound infection that may occur following resection of the corrugator supercilii muscle are among the common reasons for repeated hospital visits. Several studies have reported that, in addition to other wound-related complications, surgical site infection is one of the leading causes of readmission within 30 days following plastic surgery interventions [22].

The frontal region is a highly preferred region today for many procedures, such as fascial rejuvenation or migraine-related muscle resections and botulinum toxin applications for wrinkle removal and trigger point relief. Following surgical interventions applied to this region, undesired muscle contraction conditions such as synkinesis may occur. Synkinesis is defined as the involuntary contraction of a muscle group accompanying the voluntary contraction of another muscle group [23].

Among the well-known types of synkinesis, Marcus Gunn jaw-winking synkinesis has been reported to occur either due to the connection between the trigeminal and oculomotor nerves [24] or as a result of injury to the buccal branch following facelift procedures [23]. A rarer form, oculonasal synkinesis, was described in detail by Guarro et al. through a video-based case series [23]. In this uncommon phenomenon, new neural connections may develop between the temporal and zygomatic branches and the buccal branch of the facial nerve following plastic surgical interventions. In such patients, alar complex contractions triggered by blinking were manageable through incision of the transverse part of nasalis (compressor narium minor) muscle [23].

## Limitations

This study utilized formalin-fixed cadavers, which may alter tissue elasticity and spatial relationships compared to living tissues. The sample size (44 hemifaces from 22 cadavers), though sufficient for morphometric analysis, may not capture all anatomical variations encountered clinically. Additionally, the exclusion of cadavers with prior trauma or surgery could limit generalizability to patients with altered frontal anatomy. Functional implications of the observed neurovascular relationships (e.g., dynamic nerve compression during muscle contraction) were not assessed, warranting further in vivo studies.

## Conclusion

**Fig. 5:**
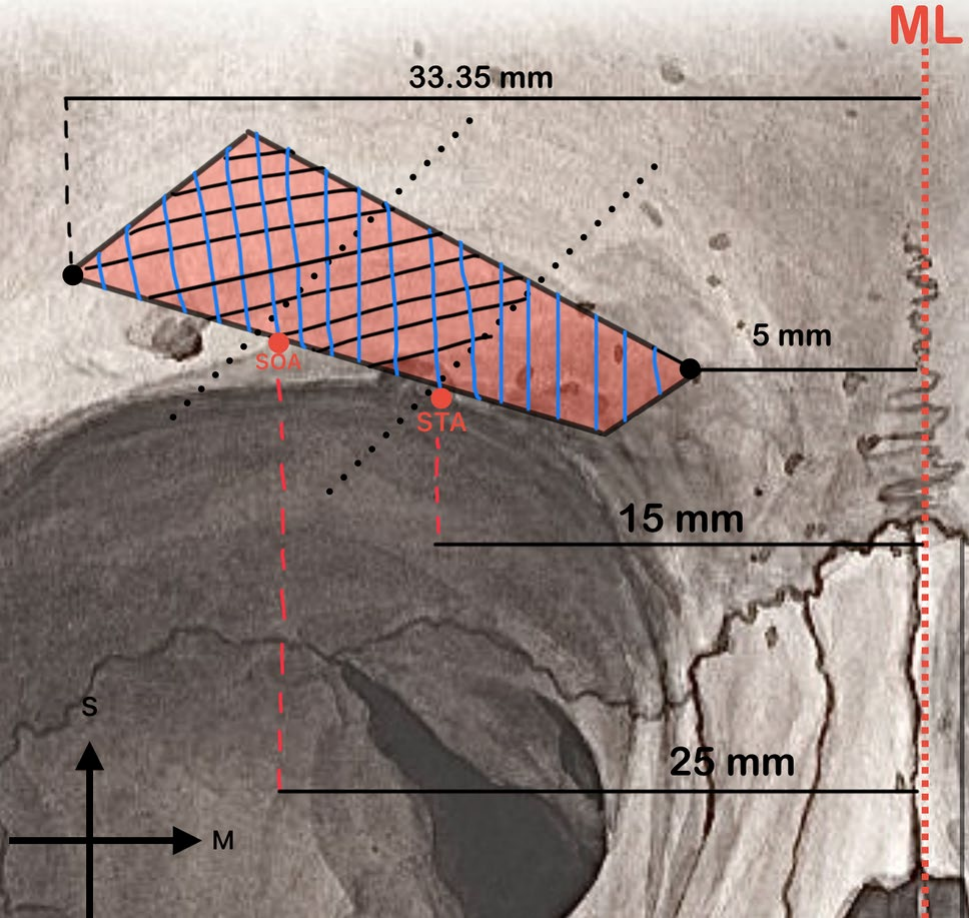
Demonstration of the arterial and neural distribution areas of the corrugator supercilii muscle and the relationship between the medial and lateral points and the midsagittal line. 
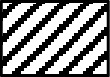
: SON distribution area (area hatched with oblique black lines). 
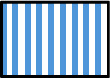
: STN distribution area (area hatched with vertical blue lines)

The origin of the CSM is located approximately 5 mm lateral to the midline in women and men (5.08 and 4.92 mm, respectively), and its length is on average 30.53 ± 2.6 mm in women and 28.93 ± 2.15 mm in men. The muscle is in close relationship with the STN and SON, which are sensory nerves in the region. The STN gives off a different number of branches that emerge from the orbit in various shapes and can extend up to the lateral 1/3 of the muscle. After emerging from a notch or foramen, the SON progresses on the back side of the muscle as the medial branch and the lateral branch. The medial branch pierces the lateral 2/3 of the muscle and passes to the anterior face, and the lateral branch crosses the muscle from its posterior, approximately 28.25 mm away from the ML, and proceeds to the frontotemporal junction on the periosteum surface. The STA crosses the inferior border of the CSM approximately 15 mm lateral to the ML. The SOA passes through the posterior aspect of the muscle, approximately 25 mm lateral to the ML. Based on the approximate distances specified, the areas within the region bounded by nerves and arteries, as well as the danger and safe zones formed by the muscle’s origin and insertion points in relation to the midline, were revealed (Fig. 5). In surgical procedures such as endoscopic brow lift and migraine surgery, where partial or complete excision of the muscle is required, these mean values may provide clinically useful reference points. Nevertheless, validation and further refinement of these findings through future large-scale and/or in vivo studies would be beneficial.

Affecting the SON and STN branches during interventional procedures applied to the area may cause undesirable sensory losses in a wide area distributed over the forehead skin, glabella, upper eyelid, and conjunctiva. Affecting STA and SOA may cause a wide spectrum of complications such as bleeding, anaphylaxis, and blindness. Failure to completely remove the muscle during resection and the presence of residual muscle around the neural structures or in the section extending laterally may reduce patient satisfaction after rejuvenation operations and migraine surgery and may cause recurrent pain.

Considering these neurovascular distributions and neighborhood relationships, it is expected that more effective and adequate treatments will be applied, and patient satisfaction will increase by reducing the risk of complications.

## Declarations

### Explanation

This study is from the medical specialization thesis in anatomy entitled “Evaluation of Corrugator Supercilii Muscle and Its Innervation In Terms of Invasive Procedures of Frontal Region” presented on 06/11/2024.
